# Change in healthcare utilization and costs following initiation of benzodiazepine therapy for long-term treatment of generalized anxiety disorder: a retrospective cohort study

**DOI:** 10.1186/1471-244X-12-177

**Published:** 2012-10-23

**Authors:** Ariel Berger, John Edelsberg, Michael Treglia, Jose Ma J Alvir, Gerry Oster

**Affiliations:** 1 Policy Analysis Inc. (PAI), Brookline, MA, USA; 2 Pfizer Inc, New York, NY, USA

**Keywords:** Anxiety Disorders, Benzodiazepines, Utilization, Costs and Cost Analysis, Healthcare Research

## Abstract

**Background:**

Selective serotonin reuptake inhibitors, serotonin-norepinephrine reuptake inhibitors, and benzodiazepine anxiolytics are used in the US to treat generalized anxiety disorder (GAD). While benzodiazepines typically provide rapid symptomatic relief, long-term use is not recommended due to risks of dependency, sedation, falls, and accidents.

**Methods:**

Using a US health insurance database, we identified all persons with GAD (ICD-9-CM diagnosis code 300.02) who began a long-term course of treatment (≥90 days) with a benzodiazepine anxiolytic between 1/1/2003 and 12/31/2007, We compared healthcare utilization and costs over the six-month periods preceding and following the date of treatment initiation (“pretreatment” and “post-treatment”, respectively), and focused attention on accident-related encounters (e.g., for treatment of fractures) and care received for other reasons possibly related benzodiazepine use (e.g., sedation, dizziness).

**Results:**

A total of 866 patients met all study entry criteria; 25% of patients began treatment on an add-on basis (i.e., adjunctive to escitalopram, paroxetine, sertraline, or venlafaxine), while 75% of patients did not receive concomitant therapy. Mean total healthcare costs increased by $2334 between the pretreatment and post-treatment periods (from $4637 [SD=$9840] to $6971 [$17,002]; p<0.01); costs of accident-related encounters and other care that was possibly related to use of benzodiazepines increased by an average of $1099 ($1757 [$7656] vs $2856 [$14,836]; p=0.03).

**Conclusions:**

Healthcare costs increase in patients with GAD beginning long-term (≥90 days) treatment with a benzodiazepine anxiolytic; a substantial proportion of this increase is attributable to care associated with accidents and other known sequelae of long-term benzodiazepine use.

## Background

Generalized anxiety disorder (GAD) is a chronic condition characterized by persistent worry or anxiety [1]; GAD is often difficult to diagnose because of the wide variety of clinical presentations and the common occurrence of comorbid somatic diseases and/or mental disorders. GAD typically follows a relapsing/remitting pattern; approximately one-third of patients who achieve remission experience a full relapse within three years [2].

Lifetime prevalence of GAD has been estimated to be about 4-6% [3]; one-year prevalence has been reported to be about 2% [4,5]. GAD is two to three times more common in women than men [4]. GAD is the most common anxiety disorder among patients presenting to primary care physicians [6,7], and it is overrepresented in primary care settings, with point prevalence rates at least 2–3 times higher than those reported in the community [7,8].

While benzodiazepines were the mainstay of GAD treatment for many years, selective serotonin reuptake inhibitors (SSRIs) and serotonin-norepinephrine reuptake inhibitors (SNRIs) currently are more frequently used as first-line treatment for this condition, as these agents are also effective against depression, which is common among patients with GAD [9]. However, even if benzodiazepines are not typically used on a first-line basis, they are often prescribed adjunctively as they typically provide rapid symptomatic relief and are relatively well tolerated. Current guidelines suggest the use of benzodiazepines as short-term concomitant therapy with recommended first-line antidepressants to speed onset of efficacy, suppression of increased anxiety associated with initiation of serotonergic therapy, and/or short-term relief for crises; long-term use of benzodiazepines is recommended for patients deemed refractory to other available therapies [10,11]. There is general agreement today that benzodiazepines should not be used for more than a few weeks due to risks of dependency and sedation, increased risk of falls, industrial and motor vehicle accidents, and neonatal and infant mortality when used in late pregnancy or during breast feeding [12-17]. A substantial proportion of patients receiving benzodiazepines also develop rebound anxiety, an intensification of previous symptoms, or withdrawal when treatment is discontinued [18-20]. These concerns are heightened in older patients, as risk of adverse events generally increases with age [12-14,21-23].

In a study using US health insurance data that compared patients with various diagnoses who were prescribed benzodiazepines (n=4454) with those who received other (i.e., non-benzodiazepine) anxiolytics (n=13,662), Oster and colleagues reported that patients in the former group had a 15% higher risk of accident-related medical events; risk was 30% higher among those who had filled three or more prescriptions for benzodiazepines than among those who had filled only one [24]. The association between use of benzodiazepines and increased risk of accident/injury (including but not limited to motor-vehicle accidents, falls, and fractures) has been confirmed in several subsequent studies [25-32]; benzodiazepines also have been associated with impairment of cognitive and physical function, dizziness, and sleep impairment [33-38]. Current treatment guidelines for GAD recommend first-line treatment with antidepressants—specifically, escitalopram, paroxetine, or sertraline (all selective serotonin re-uptake inhibitors [SSRI]), or venlafaxine (a serotonin-norepinephrine re-uptake inhibitor [SNRI])—or pregabalin on the basis of their efficacy, safety, and tolerability [10,11,39].

While prior research has established the association between use of benzodiazepines and increased risk of various adverse events, most of such work has focused attention on older adults. Moreover, relatively little is known about the extent to which these risks are experienced among patients with GAD—a condition for which benzodiazepines often are prescribed. To build upon prior research, in this study we used data from a large US healthcare claims database to examine changes in healthcare utilization and costs in patients with GAD beginning a long-term (≥90 days) course of treatment with a benzodiazepine anxiolytic.

## Methods

### Data source

Data were obtained from the PharMetrics Patient-Centric Database, which is comprised of facility, professional-service, and retail (i.e., outpatient) pharmacy claims from over 85 US health plans. The plans provide healthcare coverage to approximately 14 million persons annually throughout the US (Midwest, 35%; Northeast, 21%; South, 31%; West, 13%). All patient identifiers in the database have been fully encrypted, and the database is fully compliant with the Health Insurance Portability and Accountability Act of 1996 (HIPAA). Since there was no contact with either patients or their providers, all patient-identifying information was de-identified, and all analyses were retrospective in nature, Institutional Review Board (IRB) approval was not required.

Information available for each facility and professional-service claim includes date and place of service, diagnoses (in ICD-9-CM format), procedures (in ICD-9-CM [selected plans only], Current Procedural Terminology, 4th Edition [CPT-4], and HCPCS formats), and provider specialty. Data available for each retail pharmacy claim include the drug dispensed (in NDC format), the dispensing date, and the quantity dispensed and number of days of therapy supplied (selected plans only). All claims include both charged and paid amounts, the latter including patient deductibles, copayments, and/or coinsurance.

Selected demographic and eligibility information is also available, including age, gender, geographic region, coverage type, and the dates of insurance coverage. All patient-level data can be arrayed in chronologic order to provide a detailed, longitudinal profile of all medical and pharmacy services used by each plan member. The database for this study encompassed the period from January 1, 2003 through December 31, 2007 (“study period”).

### Study sample

We identified all patients with two or more paid claims for outpatient encounters on different days with a diagnosis of GAD (ICD-9-CM diagnosis code 300.02) between 1/1/2003 and 12/31/2007. Among these persons, we limited attention to those who were beginning a long-term course of treatment (≥90 therapy-days over 6 months) with a benzodiazepine anxiolytic (i.e., alprazolam, chlordiazepoxide, clonazepam, clorazepate, diazepam, lorazepam, oxazepam) (“study agents”). Date of treatment initiation was designated the “index date”. We excluded from the study sample all patients: (1) with any evidence of receipt of benzodiazepines prior to their index date; (2) with one or more days of ineligibility for medical and pharmacy benefits over the 6-month periods immediately preceding and following their index dates (“pretreatment” and “post-treatment”, respectively); (3) who were Medicaid beneficiaries; or (4) aged ≥65 years as of their index date and enrolled in a Medicare supplemental or fee-for-service plan (their claims histories may be incomplete). We then stratified patients according to whether they received a benzodiazepine anxiolytic alone (“monotherapy cohort”) or adjunctive to escitalopram, paroxetine, sertraline, or venlafaxine (“add-on” cohort), as described below.

To maximize the likelihood that patients in the monotherapy cohort were initiating treatment for GAD, we required that they have at least one healthcare encounter with a diagnosis code of GAD in the 90-day period immediately preceding (and including) their index date, and no evidence of receipt of any other GAD-related medication (i.e., escitalopram, paroxetine, sertraline, venlafaxine, imipramine, buspirone, hydroxyzine, trifluoperazine) [9,40] or any other agent from any corresponding class of medication (e.g., selective serotonin reuptake inhibitors [SSRIs]) during the 6-month pretreatment period.

Patients in the add-on cohort were required to have at least one claim with a diagnosis code of GAD as well as evidence of extended (≥90 days) receipt of escitalopram, paroxetine, sertraline, or venlafaxine (all recommended as first-line treatment for GAD in recent clinical guidelines [9,41]) during the 6-month period preceding initiation of benzodiazepine therapy (a longer time period was used for the add-on cohort than the monotherapy cohort, as GAD diagnoses may not be rendered as frequently in patients receiving long-term therapy). They also had to have evidence of receipt of these agents in the 90-day period following their index date.

Patients not meeting criteria for either the monotherapy or add-on cohorts were dropped from the study sample. For all remaining patients, we compiled all pharmacy, professional service, and facility claims during both the pre-index and post-index periods.

### Measures and analyses

The demographic and clinical characteristics of study subjects, including prevalence of selected comorbidities (Appendix), were characterized on the basis of information during the 6-month pre-index period.

All healthcare encounters with ICD-9-CM diagnosis codes in the ranges 800.XX-959.XX, 990.XX-995.XX, E800.X-E849.X, E880.X-E889.X, E916.X-E929.X, and V155.5 were considered to be “accident-related,” following methods first employed by Oster *et al.*[24]. Healthcare encounters for other potential adverse consequences of long-term benzodiazepine therapy were identified based on visits/admissions for known sequelae of benzodiazepine use [33–38], as follows: (1) drug dependence/addiction/poisoning (ICD-9-CM diagnosis codes 304.1X, 304.9X, 305.4, 969.4, V58.69, E853.2, E980.3); (2) withdrawal syndrome (292.0X); (3) drowsiness (780.09); (4) ataxia (781.2, 781.3); (5) dysarthria (784.5); (6) diplopia (368.2); (7) vertigo/dizziness (780.4); (8) mental confusion/disorientation (292.8X, 292.9X, 293.0, 780.1, 780.2); (9) cognitive impairment (780.02, 780.09, 780.93, 780.97); and (10) other adverse effects recorded that related to benzodiazepine therapy (E939.4, E950.3). These encounters were denoted, collectively, as “other possibly related” care.

We also examined patterns of healthcare utilization during the pretreatment and post-treatment periods in terms of the numbers of physician office visits, other outpatient office visits, emergency department (ED) visits, and hospitalizations. Use of these services was examined in terms of the numbers of patients receiving each type of service, as well as counts of the numbers of ervices used (e.g., office visits, hospitalizations). All healthcare encounters and corresponding costs were designated as “accident-related”, “other possibly related”, or “other”, depending on the ICD-9-CM diagnosis code(s) noted on each claim. Total healthcare costs were tallied in terms of: (1) prescription pharmacotherapy; (2) physician office visits; (3) other outpatient visits; (4) ED visits; (5) inpatient care; and (6) all other care. Reimbursed amounts (including any patient liability, such as co-pays and co-insurance) were used in all analyses of healthcare costs. Healthcare utilization and costs were assessed over the 6-month pretreatment and post-treatment periods, respectively.

Since patients served as their own controls in the analyses, the statistical significance of differences in healthcare costs between the pretreatment and post-treatment periods was assessed using Wilcoxon signed-rank tests; McNemar and Bowker’s tests were used to assess the statistical significance of differences in categorical variables. All tests of statistical significance were two-tailed with an alpha level of 0.05. All analyses were first performed by cohort and, within each cohort, on an overall basis as well as for patients aged <50 years versus ≥50 years, respectively. Finally, analyses were performed for the two cohorts combined, on an overall basis as well as by age stratum (<50 years vs ≥50 years). All analyses were conducted using SAS® Proprietary Software, Release 9.1 (SAS Institute Inc., Cary, NC).

## Results

We identified a total of 866 patients with GAD who began a long-term course of treatment (≥90 days) with a benzodiazepine anxiolytic; 25% of patients began treatment on an add-on basis (i.e., adjunctive to escitalopram, paroxetine, sertraline, or venlafaxine), while 75% of patients did not receive concomitant therapy. Mean (SD) age was 43.3 (13.6) years, and 53.3% were women (Table 1). Forty-five percent of study subjects had evidence of comorbid depression. Mean age was similar in the monotherapy and add-on cohorts (about 43 years); the latter were more likely to be women, however (65% vs 50% of those in the monotherapy cohort), and to have comorbid depression (61% vs 40%) (data available upon request).

**Table 1:** Demographic and clinical characteristics of GAD patients designated as long-term benzodiazepine users, by age

**Characteristic**	**Patient Age**		**All Patients (N=866)**
	**<50 Years (N=568)**	**≥50 Years (N=298)**	
Mean (SD) age, years	35.8 (9.7)	57.6 (7.1)	43.3 (13.6)
Women	293 (51.6)	169 (56.7)	462 (53.3)
Comorbidities			
Mental disorders			
Other anxiety disorders	105 (18.5)	43 (14.4)	148 (17.1)
Depressive disorders	259 (45.6)	133 (44.6)	392 (45.3)
Bipolar disorder	9 (1.6)	5 (1.7)	14 (1.6)
Tension headache	6 (1.1)	2 (0.7)	8 (0.9)
Personality disorders	7 (1.2)	4 (1.3)	11 (1.3)
Alcohol abuse/alcoholism	3 (0.5)	2 (0.7)	5 (0.6)
Drug abuse	15 (2.6)	2 (0.7)	17 (2.0)
Suicide attempts	13 (2.3)	5 (1.7)	18 (2.1)
All other			
Sleep disorders	65 (11.4)	47 (15.8)	112 (12.9)
Neoplasms	8 (1.4)	15 (5.0)	23 (2.7)
Diabetes	12 (2.1)	36 (12.1)	48 (5.5)
Migraine	17 (3.0)	10 (3.4)	27 (3.1)
Ischemic heart disease	2 (0.4)	14 (4.7)	16 (1.8)
Cerebrovascular disease	6 (1.1)	8 (2.7)	14 (1.6)
Asthma	23 (4.0)	19 (6.4)	42 (4.8)
Painful neuropathic disorders	54 (9.5)	45 (15.1)	99 (11.4)
Symptoms, signs, and ill-defined conditions			
Fatigue	71 (12.5)	45 (15.1)	116 (13.4)
Headache	50 (8.8)	20 (6.7)	70 (8.1)
Chest pain	55 (9.7)	31 (10.4)	86 (9.9)
Abdominal pain	65 (11.4)	31 (10.4)	96 (11.1)
Anxiety-related symptoms	72 (12.7)	48 (16.1)	120 (13.9)
Any symptoms, signs, and ill-defined conditions	211 (37.1)	119 (39.9)	330 (38.1)
Region			
Northeast	164 (28.9)	84 (28.2)	248 (28.6)
South	140 (24.6)	54 (18.1)	194 (22.4)
West	92 (16.2)	55 (18.5)	147 (17.0)
Midwest	172 (30.3)	105 (35.2)	277 (32.0)
Payer type			
HMO	138 (24.3)	63 (21.1)	201 (23.2)
PPO	300 (52.8)	152 (51.0)	452 (52.2)
Indemnity	25 (4.4)	22 (7.4)	47 (5.4)
Other	33 (5.8)	19 (6.4)	52 (6.0)

*Unless otherwise noted, all values are numbers of patients (%).

GAD: Generalized anxiety disorder; HMO: Health maintenance organization; PPO: Preferred provider organization.

Changes in the number of persons with accident-related and other possibly related encounters between the pretreatment and post-treatment periods were minor and not statistically significant, irrespective of age (Table 2). Mean numbers of outpatient visits for accident-related and other possibly related care increased significantly between the two periods, however (from 6.1 [17.3] to 8.0 [20.2]; p<0.01).

**Table 2:** Use of healthcare services during pre- and post-index periods among all GAD patients newly started on benzodiazepines, by age*

**Healthcare Service**	**Patient Age**						**All Patients (N=866)**		
	**<50 Years (N=568)**			**≥50 Years (N=298)**					
	**Pre-Index**	**Post-Index**	***P*-Value**	**Pre-Index**	**Post-Index**	***P*-Value**	**Pre-Index**	**Post-Index**	***P*-Value**
Outpatient care									
Outpatient office visits									
Number (%) with ≥1 visits									
Accident-related	102 (18.0)	106 (18.7)	0.72	51 (17.1)	53 (17.8)	0.80	153 (17.7)	159 (18.4)	0.66
Other possibly related	60 (10.6)	69 (12.1)	0.33	37 (12.4)	32 (10.7)	0.47	97 (11.2)	101 (11.7)	0.73
All of above	142 (25.0)	152 (26.8)	0.45	79 (26.5)	73 (24.5)	0.51	221 (25.5)	225 (26.0)	0.80
All other	379 (66.7)	410 (72.2)	0.03	209 (70.1)	223 (74.8)	0.14	588 (67.9)	633 (73.1)	<0.01
All	521 (91.7)	562 (98.9)	<0.01	288 (96.6)	296 (99.3)	0.02	809 (93.4)	858 (99.1)	<0.01
Mean (SD) number of visits									
Accident-related	4.2 (14.3)	5.3 (15.0)	0.15	5.9 (19.9)	6.4 (19.2)	0.69	4.8 (16.4)	5.7 (16.5)	0.18
Other possibly related	2.5 (11.2)	3.6 (12.8)	0.06	3.0 (12.1)	5.7 (23.9)	0.05	2.7 (11.5)	4.3 (17.4)	<0.01
All of above	5.5 (15.3)	7.2 (16.6)	0.02	7.4 (20.5)	9.6 (25.7)	0.11	6.1 (17.3)	8.0 (20.2)	<0.01
All other	8.0 (13.7)	11.6 (15.9)	<0.01	12.8 (24.3)	17.7 (24.7)	<0.01	9.6 (18.2)	13.7 (19.6)	<0.01
All	13.4 (18.2)	18.8 (19.1)	<0.01	20.2 (28.7)	27.3 (30.5)	<0.01	15.8 (22.6)	21.7 (23.9)	<0.01
ED visits									
Number (%) with ≥1 visits									
Accident-related	44 (7.7)	48 (8.5)	0.60	15 (5.0)	15 (5.0)	>0.99	59 (6.8)	63 (7.3)	0.67
Other possibly related	36 (6.3)	23 (4.0)	0.06	9 (3.0)	14 (4.7)	0.28	45 (5.2)	37 (4.3)	0.33
All of above	67 (11.8)	61 (10.7)	0.51	21 (7.0)	24 (8.1)	0.62	88 (10.2)	85 (9.8)	0.79
All other	51 (9.0)	58 (10.2)	0.44	18 (6.0)	24 (8.1)	0.29	69 (8.0)	82 (9.5)	0.23
All	118 (20.8)	119 (21.0)	0.93	39 (13.1)	48 (16.1)	0.26	157 (18.1)	167 (19.3)	0.48
Mean (SD) number of visits									
Accident-related	0.7 (3.7)	1.0 (6.1)	0.27	0.6 (3.4)	1.3 (15.5)	0.47	0.7 (3.6)	1.1 (10.3)	0.25
Other possibly related	0.8 (4.8)	0.7 (5.5)	0.47	1.1 (15.1)	1.4 (15.6)	0.24	0.9 (9.6)	0.9 (10.2)	0.92
All of above	1.2 (5.2)	1.3 (6.5)	0.88	1.6 (15.4)	1.6 (15.7)	0.81	1.4 (9.9)	1.4 (10.6)	0.98
All other	0.8 (4.0)	1.0 (4.7)	0.43	0.6 (3.1)	0.8 (4.0)	0.51	0.7 (3.7)	0.9 (4.5)	0.30
All	2.1 (6.4)	2.3 (7.9)	0.49	2.2 (15.6)	2.3 (16.1)	0.72	2.1 (10.5)	2.3 (11.4)	0.44
Hospitalizations									
Number (%) with ≥1 hospitalizations									
Accident-related	11 (1.9)	13 (2.3)	0.67	8 (2.7)	7 (2.3)	0.76	19 (2.2)	20 (2.3)	0.86
Other possibly related	12 (2.1)	13 (2.3)	0.83	8 (2.7)	10 (3.4)	0.56	20 (2.3)	23 (2.7)	0.60
All of above	18 (3.2)	22 (3.9)	0.50	12 (4.0)	11 (3.7)	0.78	30 (3.5)	33 (3.8)	0.67
All other	23 (4.0)	17 (3.0)	0.29	14 (4.7)	11 (3.7)	0.44	37 (4.3)	28 (3.2)	0.19
All	41 (7.2)	39 (6.9)	0.80	26 (8.7)	22 (7.4)	0.41	67 (7.7)	61 (7.0)	0.51
Mean (SD) number of hospitalizations									
Accident-related	0.7 (6.1)	1.2 (11.2)	0.38	1.1 (8.6)	1.3 (10.1)	0.75	0.9 (7.0)	1.2 (10.8)	0.36
Other possibly related	0.9 (7.3)	1.3 (12.1)	0.52	1.5 (10.9)	1.7 (11.0)	0.74	1.1 (8.7)	1.4 (11.7)	0.48
All of above	1.2 (8.1)	1.7 (13.0)	0.40	1.7 (11.1)	1.8 (11.0)	0.98	1.4 (9.3)	1.7 (12.4)	0.47
All other	0.9 (5.9)	0.5 (4.1)	0.13	1.7 (9.0)	0.8 (5.9)	0.11	1.2 (7.1)	0.6 (4.8)	0.03
All	2.2 (9.9)	2.2 (13.6)	0.94	3.5 (14.1)	2.6 (12.4)	0.30	2.6 (11.6)	2.3 (13.2)	0.56

GAD: Generalized anxiety disorder; SD: Standard deviation; ED: Emergency department.

Mean (SD) total healthcare costs increased by an average of $2334 between the pretreatment and post-treatment periods ($4637 [$9840] vs $6971 [$17,002]; p<0.01); the difference in median costs was $1414 ($1805 during pre-treatment period vs $3219 for during post-treatment period) (Table 3). Total healthcare costs increased by $2980 among patients aged ≥50 years ($6211 [$13,308] vs 9191 [22,540]; p=0.01), and by $1994 among patients aged <50 years ($3812 [$7278] vs $5806 [$13,073]; p<0.01); corresponding median increases were $1623 (median [IQR] = $2381 [$816, $6017] vs $4004 [$1886, $8409]) and $1285 ($1540 [$495, $3723] vs $2825 [$1304, $5767]). Mean costs for accident-related and other possibly related care increased by $1099 between the two periods ($1757 [$7656] vs $2856 [$15,742]; p=0.03). Patients aged ≥50 years experienced nominally greater increases in these costs than those who were younger ($1559 and $858, respectively).

**Table 3:** Total healthcare costs during pre- and post-index periods among al l GAD patients newly started on benzodiazepines, by age*

	**Patient Age**						**All Patients (N=866)**		
	**<50 Years (N=568)**			**≥50 Years (N=298)**					
	**Pre-Index**	**Post-Index**	**P-Value**	**Pre-Index**	**Post-Index**	**P-Value**	**Pre-Index**	**Post-Index**	**P-Value**
Pharmacotherapy									
Mean (SD)	583 (1,660)	1,295 (1,789)	<0.01	829 (1,446)	1,873 (2,318)	<0.01	668 (1,593)	1,494 (2,005)	<0.01
Median (IQR)	154 (0, 661)	813 (450, 1,497)	---	397 (17, 1,061)	1,217 (640, 2,165)	---	215 (3, 817)	917 (483, 1,695)	---
Outpatient services									
Outpatient office visits									
Accident-related									
Mean (SD)	437 (2,368)	518 (1,757)	0.46	591 (2,203)	749 (2,687)	0.27	490 (2,312)	598 (2,125)	0.22
Median (IQR)	0 (0, 0)	0 (0, 0)	---	0 (0, 0)	0 (0, 0)	---	0 (0, 0)	0 (0, 0)	---
Other possibly related									
Mean (SD)	287 (2,101)	418 (1,695)	0.19	297 (1,627)	624 (2,901)	0.04	290 (1,950)	489 (2,187)	0.02
Median (IQR)	0 (0, 0)	0 (0, 0)	---	0 (0, 0)	0 (0, 0)	---	0 (0, 0)	0 (0, 0)	---
All of above									
Mean (SD)	552 (2,416)	766 (2,095)	0.07	701 (2,221)	1,099 (3,425)	0.03	603 (2,351)	881 (2,633)	<0.01
Median (IQR)	0 (0, 42)	0 (0, 422)	---	0 (0, 78)	0 (0, 0)	---	0 (0, 78)	0 (0, 315)	---
All other									
Mean (SD)	802 (2,300)	1,228 (2,913)	<0.01	1,331 (2,939)	1,840 (3,502)	0.03	984 (2,549)	1,439 (3,140)	<0.01
Median (IQR)	211 (0, 822)	432 (0, 1,300)	---	395 (0, 1,368)	678 (0, 2,061)	---	247 (0, 985)	483 (0, 1,565)	---
All									
Mean (SD)	1,354 (3,201)	1,995 (3,315)	<0.01	2,032 (3,421)	2,938 (4,465)	<0.01	1,587 (3,292)	2,319 (3,775)	<0.01
Median (IQR)	533 (187, 1,455)	993 (430, 2,239)	---	845 (363, 2,174)	1,520 (548, 3,274)	---	641 (221, 1,623)	1,171 (463, 2,563)	---
ED visits									
Accident-related									
Mean (SD)	99 (538)	143 (934)	0.16	59 (384)	161 (2,004)	0.39	85 (490)	149 (1,397)	0.16
Median (IQR)	0 (0, 0)	0 (0, 0)	---	0 (0, 0)	0 (0, 0)	---	0 (0, 0)	0 (0, 0)	---
Other possibly related									
Mean (SD)	116 (708)	99 (838)	0.57	96 (1,328)	178 (2,021)	0.08	109 (966)	126 (1,365)	0.50
Median (IQR)	0 (0, 0)	0 (0, 0)	---	0 (0, 0)	0 (0, 0)	---	0 (0, 0)	0 (0, 0)	---
All of above									
Mean (SD)	173 (768)	180 (976)	0.84	150 (1,377)	207 (2,036)	0.23	165 (1,019)	189 (1,431)	0.40
Median (IQR)	0 (0, 0)	0 (0, 0)	---	0 (0, 0)	0 (0, 0)	---	0 (0, 0)	0 (0, 0)	---
All other									
Mean (SD)	105 (547)	122 (554)	0.57	75 (440)	111 (677)	0.42	94 (513)	118 (599)	0.35
Median (IQR)	0 (0, 0)	0 (0, 0)	---	0 (0, 0)	0 (0, 0)	---	0 (0, 0)	0 (0, 0)	---
All									
Mean (SD)	278 (924)	302 (1,103)	0.60	225 (1,438)	317 (2,135)	0.15	259 (1,127)	307 (1,537)	0.20
Median (IQR)	0 (0, 0)	0 (0, 0)	---	0 (0, 0)	0 (0, 0)	---	0 (0, 0)	0 (0, 0)	---
Hospitalizations									
Accident-related									
Mean (SD)	357 (3,235)	873 (10,042)	0.24	871 (7,386)	1,660 (17,048)	0.42	534 (5,065)	1,144 (12,885)	0.17
Median (IQR)	0 (0, 0)	0 (0, 0)	---	0 (0, 0)	0 (0, 0)	---	0 (0, 0)	0 (0, 0)	---
Other possibly related									
Mean (SD)	341 (2,898)	778 (9,468)	0.28	1,031 (8,303)	2,025 (17,635)	0.33	578 (5,412)	1,207 (12,880)	0.16
Median (IQR)	0 (0, 0)	0 (0, 0)	---	0 (0, 0)	0 (0, 0)	---	0 (0, 0)	0 (0, 0)	---
All of above									
Mean (SD)	508 (3,650)	1,131 (10,472)	0.17	1,202 (8,516)	2,090 (17,663)	0.39	747 (5,809)	1,461 (13,387)	0.13
Median (IQR)	0 (0, 0)	0 (0, 0)	---	0 (0, 0)	0 (0, 0)	---	0 (0, 0)	0 (0, 0)	---
All other									
Mean (SD)	444 (2,655)	252 (2,255)	0.18	865 (5,774)	396 (2,906)	0.20	589 (4,014)	301 (2,498)	0.07
Median (IQR)	0 (0, 0)	0 (0, 0)	---	0 (0, 0)	0 (0, 0)	---	0 (0, 0)	0 (0, 0)	---
All									
Mean (SD)	953 (4,463)	1,382 (10,685)	0.35	2,067 (10,187)	2,485 (17,854)	0.70	1,336 (6,998)	1,762 (13,585)	0.38
Median (IQR)	0 (0, 0)	0 (0, 0)	---	0 (0, 0)	0 (0, 0)	---	0 (0, 0)	0 (0, 0)	---
All other care									
Accident-related									
Mean (SD)	80 (542)	78 (712)	0.95	124 (596)	247 (1,654)	0.18	95 (561) 136	(1,131)	0.30
Median (IQR)	0 (0, 0)	0 (0, 0)	---	0 (0, 0)	0 (0, 0)	---	0 (0, 0)	0 (0, 0)	---
Other possibly related									
Mean (SD)	63 (353)	55 (326)	0.65	100 (606)	267 (2,364)	0.23	76 (456)	128 (1,414)	0.29
Median (IQR)	0 (0, 0)	0 (0, 0)	---	0 (0, 0)	0 (0, 0)	---	0 (0, 0)	0 (0, 0)	---
All of above									
Mean (SD)	118 (599)	118 (759)	>0.99	177 (754)	423 (2,772)	0.13	138 (657)	223 (1,743)	0.16
Median (IQR)	0 (0, 0)	0 (0, 0)	---	0 (0, 0)	0 (0, 0)	---	0 (0, 0)	0 (0, 0)	---
All other									
Mean (SD)	154 (540)	253 (1,405)	0.07	381 (1,535)	544 (2,845)	0.32	232 (1,006)	353 (2,023)	0.07
Median (IQR)	0 (0, 61)	0 (0, 63)	---	4 (0, 175)	9 (0, 224)	---	0 (0, 98)	0 (0, 106)	---
All									
Mean (SD)	272 (784)	371 (1,578)	0.13	559 (1,670)	967 (3,913)	0.06	370 (1,174)	576 (2,640)	0.02
Median (IQR)	16 (0, 191)	22 (0, 164)	---	84 (0, 374)	91 (0, 424)	---	37 (0, 237)	35 (0, 247)	---
Total									
Accident-related									
Mean (SD)	1,025 (4,833)	1,662 (11,324)	0.20	1,787 (9,131)	2,947 (19,831)	0.26	1,287 (6,638)	2,104 (14,814)	0.09
Median (IQR)	0 (0, 0)	0 (0, 0)	---	0 (0, 0)	0 (0, 0)	---	0 (0, 0)	0 (0, 0)	---
Other possibly related									
Mean (SD)	835 (4,628)	1,381 (10,518)	0.21	1,594 (10,275)	3,174 (21,009)	0.17	1,096 (7,101)	1,998 (14,993)	0.07
Median (IQR)	0 (0, 0)	0 (0, 0)	---	0 (0, 0)	0 (0, 0)	---	0 (0, 0)	0 (0, 0)	---
All of above									
Mean (SD)	1,414 (5,387)	2,272 (11,880)	0.09	2,411 (10,709)	3,970 (21,224)	0.16	1,757 (7,656)	2,856 (15,742)	0.03
Median (IQR)	0 (0, 148)	0 (0, 539)	---	0 (0, 295)	0 (0, 0)	---	0 (0, 217)	0 (0, 397)	---
All other									
Mean (SD)	2,247 (5,378)	3,122 (6,209)	<0.01	3,505 (8,635)	4,664 (7,608)	0.04	2,680 (6,702)	3,653 (6,759)	<0.01
Median (IQR)	533 (0, 2,122)	1,343 (0, 3,521)	---	943 (0, 3,575)	2,263 (183, 5,746)	---	635 (0, 2,519)	1,528 (0, 4,236)	---
All									
Mean (SD)	3,812 (7,278)	5,806 (13,073)	<0.01	6,211 (13,308)	9,191 (22,540)	0.01	4,637 (9,840)	6,971 (17,002)	<0.01
Median (IQR)	1,540 (495, 3,723)	2,825 (1,304, 5,767)	---	2,381 (816, 6,017)	4,004 (1,886, 8,409)	---	1,805 (584, 4,590)	3,219 (1,460, 6,654)	---

*All values are healthcare costs ($).

GAD: Generalized anxiety disorder; SD: Standard deviation; ED: Emergency department; IQR: Interquartile range.

Findings with respect to accident-related and other possibly related care were similar for patients in the monotherapy and add-on cohorts. Among patients in the monotherapy cohort, mean total healthcare costs increased from $3753 ($9675) during the pretreatment period to $6073 ($15,714) during the post-treatment period (p<0.01) (median cost increased by $1404). For patients in the add-on cohort, mean total healthcare costs increased from $7396 ($9858) during the pretreatment period to $9746 ($20,241) during the post-treatment period (p=0.06) (median cost increased by $1524). Mean costs of accident-related and other possibly related care increased by $1116 among patients in the monotherapy cohort (from $1,794 [$8426] to $2910 [$14,836]; p=0.04), and by $1050 for those in the add-on cohort (from $1675 [$4574] to $2726 [$18,283]; p=0.37).

## Discussion

In our study, patients with GAD who began a long-term (≥90 days) course of therapy with a benzodiazepine anxiolytic had significantly higher healthcare costs during the 6-month period following initiation of therapy than in the 6 months preceding it. This finding is not unexpected, since initiation of benzodiazepine therapy is undoubtedly a marker for exacerbation of GAD and thus may foretell higher costs associated with the diagnosis and treatment of various associated psychic and somatic conditions. In two previous studies, we found that total healthcare costs were significantly higher following initiation of pharmacotherapy for GAD (either on a first-line basis or as add-on treatment) [42,43]. It is notable that in our current study almost one-half the increase in healthcare costs (47%) was attributable to medical encounters for reasons related to well-known side effects of long-term use of benzodiazepine anxiolytics (e.g., falls and other accidents, somnolence, vertigo, dizziness, and cognitive impairment).

Our study has a number of limitations. First, as with all studies based on healthcare claims databases, there may be errors of omission and commission in coding. Accordingly, some patients with GAD may not have been included in our study sample due to absence of appropriate and/or misdiagnoses on healthcare claims, while others who received a diagnosis of GAD incorrectly and therefore should have been excluded were not. As patient medical records were not available to us, the degree to which patients were actually misclassified is unknown. Also, the database does not contain important clinical information on disease severity. Such information, if present, might have permitted further analysis of the overall cost increase. Another potential limitation was the omission of fluoxetine from the group of SSRIs qualifying patients for the add-on group. We excluded fluoxetine because it is not indicated for the treatment of GAD, and because it has not been studied extensively in this indication. Nonetheless, there is evidence that it may be widely prescribed for this disorder [44-46]. In one recent study of 305 patients with GAD and 232 with social phobia, it was reported to be the most commonly prescribed SSRI [47]. Unless, however, patients receiving benzodiazepines as an adjunct to fluoxetine differ critically from those adding on to the other SSRIs, this omission would not have biased our study. Our findings are based on retrospective analyses of a US health insurance database; the degree to which results reported herein are generalizable to the experience of patients with GAD in other countries, where the medications indicated for the treatment GAD may differ from that in the US, is unknown. Finally, numerous p-values were calculated in our study, and we made no attempt to adjust for “multiplicity”. Thus, borderline p-values (e.g., 0.03, 0.04) should be interpreted cautiously. On the other hand, the costs increases we found were substantial, while the number of study subjects was relatively small and the standard deviations of the cost increases were relatively large. P-values that were borderline in this study would almost certainly have been unequivocally significant in a larger study.

## Conclusions

In conclusion, healthcare costs increased in patients with GAD following initiation of a long-term course of therapy with a benzodiazepine anxiolytic and approximately one-half of this increase was attributable to care associated with known sequelae of long-term use of such therapy. Thus our results appear to support the need for consideration of alternative treatment options for GAD that are relatively free of the bothersome side effects and potential for abuse associated with benzodiazepines.

## Competing interest

Mr. Berger, Dr. Edelsberg, and Dr. Oster are employed by Policy Analysis Inc., an independent contract research organization with previous and ongoing engagements with Pfizer Inc. as well as other pharmaceutical manufacturers. Drs. Treglia and Alvir are employed by Pfizer Inc. Policy Analysis Inc. received financial support from Pfizer Inc. for the conduct of this analysis and development of this manuscript. Pfizer reviewed the study research plan and the study manuscript; data management, processing, and analyses were conducted by Policy Analysis Inc.

## Authors' contributions

All authors reviewed and contributed to the study research plan, interpretation of the data, and the study manuscript; data management, processing, and analyses were conducted by AB, JE, and GO. All authors read and approved the final manuscript.

## Financial support

Funding for this research was provided by Pfizer Inc., New York, NY.

## Pre-publication history

The pre-publication history for this paper can be accessed here:

http://www.biomedcentral.com/1471-244X/12/177/prepub
